# Comparative *in vitro* antibacterial efficacy of *Melaleuca alternifolia* and *Copaifera officinalis* essential oils against multidrug-resistant bovine mastitis pathogens

**DOI:** 10.14202/vetworld.2026.2371-2378

**Published:** 2026-06-10

**Authors:** Marcela Calciolari Branquinho, Margarete Kimie Falbo, Itacir Eloi Sandini, Marina Szychta, Kate Aparecida Buzi

**Affiliations:** 1 Postgraduate Program in Veterinary Sciences, Universidade Estadual do Centro-Oeste (UNICENTRO), Guarapuava, Paraná, Brazil; 2 Department of Veterinary Medicine, Universidade Estadual do Centro-Oeste (UNICENTRO), Guarapuava, Paraná, Brazil

**Keywords:** antimicrobial resistance, bovine mastitis, *Copaifera officinalis*, essential oils, *Melaleuca alternifolia*, minimum bactericidal concentration, minimum inhibitory concentration, *Streptococcus uberis*

## Abstract

**Background and Aim:**

Bovine mastitis remains a major constraint in dairy production, leading to substantial economic losses, compromised animal welfare, and increased antimicrobial use. The emergence of multidrug-resistant (MDR) pathogens has reduced therapeutic efficacy and intensified the search for alternative antimicrobial strategies. Essential oils (EOs) have gained attention due to their bioactive properties and potential role as adjunct or alternative therapeutics. This study aimed to evaluate the *in vitro* antibacterial activity of *Melaleuca alternifolia* and *Copaifera officinalis* EOs against reference strains and clinically characterized mastitis-associated bacteria.

**Materials and Methods:**

Reference strains *Escherichia coli* American Type Culture Collection (ATCC) 25922, *Klebsiella pneumoniae* ATCC 13883, *Staphylococcus aureus* ATCC 25923, and methicillin-resistant *S. aureus* ATCC 43300, along with clinical isolates of *Streptococcus agalactiae* and *Streptococcus uberis*, were used. Antimicrobial susceptibility was assessed using the Kirby–Bauer disk diffusion method following Clinical and Laboratory Standards Institute guidelines. Minimum inhibitory concentration (MIC) and minimum bactericidal concentration (MBC) were determined by broth microdilution. EOs were tested over a concentration range of 0.49–250 mg/mL, and bactericidal activity was defined as an MBC/MIC ratio ≤ 4.

**Results:**

The antimicrobial susceptibility profiles confirmed MDR phenotypes in methicillin-resistant *S. aureus* ATCC 43300 and the clinical isolate of *S. agalactiae*. M. alternifolia EO exhibited broader antibacterial activity, inhibiting *E. coli* ATCC 25922 (MIC = 10 mg/mL; MBC/MIC = 1) and showing inhibitory effects against *S. uberis* (MIC = 15 mg/mL) and *S. agalactiae* (MIC = 31 mg/mL). However, bactericidal activity against streptococcal isolates was not observed (MBC/MIC > 4). *C. officinalis* EO demonstrated limited antibacterial activity, with growth inhibition detected only against *S. uberis* (MIC = 10 mg/mL) and *S. agalactiae* (MIC = 7.5 mg/mL), without bactericidal effects. No activity was observed against *K. pneumoniae* or methicillin-resistant *S. aureus* within the tested range.

**Conclusion:**

*M. alternifolia* EO exhibited broader antibacterial activity than *C. officinalis*, particularly against streptococcal mastitis pathogens. Although bactericidal effects were limited, the observed inhibitory activity highlights the potential of EOs as adjunct antimicrobial agents. Further *in vivo* studies, formulation optimization, and investigation of synergistic interactions with conventional antimicrobials are warranted to support their application in bovine mastitis management.

## INTRODUCTION

Bovine mastitis remains one of the most economically significant diseases in dairy production, compromising animal welfare, reducing milk yield and quality, and increasing treatment and culling costs worldwide [1]. The condition results from inflammation of the mammary gland caused primarily by infectious agents, especially bacteria classified as contagious pathogens, such as *Staphylococcus aureus*, *Streptococcus agalactiae*, *Streptococcus dysgalactiae*, *Corynebacterium bovis*, and *Mycoplasma* spp., or environmental pathogens, including *Escherichia coli*, *Klebsiella* spp., and *Pseudomonas aeruginosa* [2, 3]. Antimicrobials remain the cornerstone of mastitis therapy; however, their extensive and often inappropriate use has contributed to the emergence of multidrug-resistant (MDR) strains, compromising therapeutic efficacy and raising concerns regarding antimicrobial stewardship in both veterinary and public health contexts [4, 5].

Natural products, particularly essential oils (EOs), have gained attention as potential alternative or complementary antimicrobial agents. EOs are complex mixtures of bioactive compounds with reported antibacterial, anti-inflammatory, and antioxidant properties [6, 7]. Although several studies have demonstrated *in vitro* inhibitory effects of EOs against mastitis-associated pathogens, many investigations rely on reference laboratory strains and do not specifically assess activity against clinically characterized MDR isolates from dairy herds. Furthermore, direct comparative evaluations of *Melaleuca alternifolia* and *Copaifera officinalis* EOs against streptococcal mastitis pathogens, especially *Streptococcus uberis* and MDR *S. agalactiae*, remain limited. This limitation is particularly relevant in Brazilian dairy production systems, where regional pathogen profiles and antimicrobial resistance patterns may significantly influence therapeutic outcomes.

Despite the growing body of literature on plant-derived antimicrobials, there is a notable lack of studies that integrate the evaluation of EOs against clinically relevant MDR isolates obtained from field conditions rather than relying solely on laboratory-adapted strains. In addition, variability in EO composition, methodological inconsistencies, and the absence of standardized comparative frameworks have limited the translational applicability of existing findings. Few studies have conducted direct comparative assessments of *M. alternifolia* and *C. officinalis* under identical experimental conditions, particularly targeting streptococcal pathogens associated with bovine mastitis. Moreover, there is insufficient characterization of both inhibitory and bactericidal activities of these EOs against MDR isolates, which is critical for determining their practical therapeutic potential in mastitis control strategies.

Therefore, this study aimed to evaluate the *in vitro* antibacterial activity of *M. alternifolia* and *C. officinalis* EOs against reference strains and phenotypically characterized clinical isolates of mastitis-associated bacteria. Specifically, the study sought to determine minimum inhibitory concentration (MIC) and minimum bactericidal concentration (MBC) values and to characterize the inhibitory and bactericidal profiles of these EOs against MDR streptococcal isolates relevant to bovine mastitis. The findings are expected to contribute to the development of alternative or adjunct therapeutic approaches for the effective management of bovine mastitis.

## MATERIALS AND METHODS

### Ethical approval

Ethical review and approval were waived for this study because no live animals were used or experimentally manipulated. The study was conducted using previously obtained bacterial isolates from clinical mastitis cases provided by a diagnostic laboratory, in accordance with institutional and national guidelines for research ethics.

### Study period and location

This study was conducted from February to December 2024 at the Clinical Pathology Laboratory, Universidade Estadual do Centro-Oeste (UNICENTRO), Guarapuava, Paraná, Brazil using bacterial isolates obtained from bovine mastitis cases in dairy herds from Paraná State, Brazil. Laboratory analyses were performed in collaboration with the Animal Products Inspection and Bacteriology Laboratories of the Universidade Estadual de Londrina (Brazil).

### Study design

This study was designed as an *in vitro* experimental investigation to evaluate the antibacterial activity of EOs against reference strains and phenotypically characterized clinical isolates of mastitis-associated bacteria. The study included antimicrobial susceptibility testing and determination of MIC and MBC.

### Bacterial strains

Reference strains *E. coli* American Type Culture Collection (ATCC) 25922, *Klebsiella pneumoniae* ATCC 13883, *S. aureus* ATCC 25923, and methicillin-resistant *S. aureus* ATCC 43300 were used. Clinical isolates of *S. agalactiae* and *S. uberis*, obtained from bovine mastitis cases in dairy herds from Paraná State, Brazil, were provided by the Animal Products Inspection and Bacteriology Laboratories of the Universidade Estadual de Londrina (Brazil).

Clinical isolates were previously identified by standardized biochemical profiling (API 20 Strep system) and hemolysis patterns on blood agar (HiMedia Laboratories, Mumbai, India). Although molecular confirmation was not performed in this study, identification followed standardized biochemical procedures routinely employed in a reference veterinary diagnostic laboratory.

All strains were stored at −80°C in tryptic soy broth supplemented with 20% (v/v) glycerol. Before testing, strains were subcultured twice on Mueller–Hinton agar (Oxoid, Basingstoke, UK), for *E. coli*, *K. pneumoniae*, and *S. aureus* or Columbia agar (HiMedia Laboratories) supplemented with 5% defibrinated sheep blood (E&O Laboratories Limited, Bonnybridge, UK) for *S. agalactiae* and *S. uberis* and incubated at 35 ± 1°C for 18–24 h.

### Antimicrobial susceptibility testing

Antimicrobial susceptibility was determined using the Kirby–Bauer disk diffusion method on Mueller–Hinton agar (Oxoid), following Clinical and Laboratory Standards Institute (CLSI) M100 guidelines [8]. For *S. agalactiae* and *S. uberis*, Mueller–Hinton agar supplemented with 5% (v/v) defibrinated sheep blood (E&O Laboratories) was used.

The antimicrobial disks (Oxoid) included ampicillin (10 µg), ceftiofur (30 µg), enrofloxacin (5 µg), gentamicin (10 µg), neomycin (30 µg), penicillin (10 IU), oxacillin (1 µg), sulfamethoxazole/ trimethoprim (25 µg), and tetracycline (30 µg).

Bacterial suspensions were prepared in sterile 0.85% saline and adjusted to 0.5 McFarland standard (approximately 1.5 × 10^8^ colony-forming units (CFU)/mL) using spectrophotometric verification (OD600 = 0.08–0.10). Plates were incubated at 35 ± 1°C for 18–24 h under aerobic conditions. Inhibition zones were measured in millimeters and interpreted according to CLSI breakpoints [8]. *E. coli* ATCC 25922 and *S. aureus* ATCC 25923 were used as quality control strains.

### EOs

Tea tree (*M. alternifolia*) and copaiba (*C. officinalis*) EOs were obtained from Via Aroma® (Porto Alegre, Brazil), certified as 100% pure and produced by steam distillation. Chemical composition was based on the manufacturer’s gas chromatography–mass spectrometry report (Table 1).

**Table 1:** Major bioactive compounds of the essential oils of *Melaleuca alternifolia* and *Copaifera officinalis.*

Essential oil	Major compounds (%)
*M. alternifolia*	Terpinen-4-ol (35.1%), γ-terpinene (17.8%), α-terpinene (9.9%)
*C. officinalis*	α-pinene (28.5%), β-thujene (17.3%), D-limonene (15.6%)

EOs were stored in amber glass vials at 4°C in the dark until used to minimize oxidation and degradation.

### MIC

MIC values were determined by broth microdilution in sterile 96-well polystyrene microplates according to CLSI M07-A11 guidelines [9], with methodological adaptations to ensure appropriate dispersion and evaluation of hydrophobic EOs.

Cation-adjusted Mueller–Hinton broth (CAMHB; Oxoid, Basingstoke, UK) was used as the test medium. EOs were emulsified in 1 % (v/v) Tween 80 (Sigma-Aldrich, St. Louis, MO, USA) to ensure stable and homogeneous dispersion in the aqueous medium.

Two-fold serial dilutions of each EO were prepared to obtain final concentrations ranging from 0.49 to 250 mg/mL in a final well volume of 100 µL. Bacterial suspensions were adjusted to 0.5 McFarland standard and subsequently diluted to achieve a final concentration of approximately 5 × 10^5^ CFU/mL in each well.

Each assay included the following controls: growth control (broth + bacterial inoculum without EO), sterility control (broth only), and treatment control (broth + EO without bacterial inoculum) to exclude contamination or intrinsic color interference. All controls were included in each independent experiment.

Plates were incubated at 35 ± 1°C for 18–24 h. After incubation, 20 µL of resazurin solution (0.01%) was added to each well and incubated for an additional 2 h. MIC was defined as the lowest concentration that prevented color change from blue to pink, indicating inhibition of bacterial metabolic activity [10]. No reference antibiotic control was included in the MIC assay because the objective was to evaluate the intrinsic antibacterial activity of the EOs rather than to establish comparative antimicrobial potency under standardized CLSI-adapted conditions.

### MBC

MBC was determined from wells showing no visible growth in the MIC assay. A 100 µL aliquot from each selected well was plated onto Plate Count Agar (Oxoid, Basingstoke, UK) and incubated at 35 ± 1°C for 24 h under aerobic conditions.

MBC was defined as the lowest EO concentration resulting in a ≥99.9% reduction in CFU compared to the initial inoculum [9]. The highest concentration evaluated in the MIC assay was 250 mg/mL.

MIC and MBC assays were performed in triplicate across three independent experiments. As MIC values are determined using two-fold serial dilutions and represent ordinal rather than continuous data, results are presented as modal values (most frequently observed concentration). Inter-assay variation remained within one two-fold dilution step, indicating high reproducibility of the measurements. When minor variation occurred, the most frequently observed value across replicates was reported. Bactericidal activity was defined as an MBC/MIC ratio ≤ 4.

## RESULTS

### Antimicrobial susceptibility profiles

The antimicrobial susceptibility profiles of the tested bacterial strains are presented in Table 2 and were interpreted according to CLSI M100 guidelines [8].

**Table 2:** Antimicrobial susceptibility of mastitis-associated bacterial strains to commonly used antibiotics.

Bacterial strain	AMP	CTF	ENR	GEN	NEO	PEN	TET	OXA	SXT
*Escherichia coli* ATCC 25922	S	S	R	R	R	–	S	–	I
*Klebsiella pneumoniae* ATCC 13883	R	S	S	S	S	–	S	–	S
*Staphylococcus aureus* ATCC 25923	S	S	R	I	I	S	I	R	R
*S. aureus* ATCC 43300	R	R	I	R	R	R	S	R	S
*Streptococcus agalactiae*	S	S	I	R	R	S	S	R	R
*S. uberis*	S	S	I	–	–	S	S	–	S

S = Susceptible, I = Intermediate, R = Resistant, – = Not tested, AMP = Ampicillin, CTF = Ceftiofur, ENR = Enrofloxacin, GEN = Gentamicin, NEO = Neomycin, PEN = Penicillin, TET = Tetracycline, OXA = Oxacillin, SXT = Sulfamethoxazole/trimethoprim

*E. coli* ATCC 25922 was susceptible to ampicillin, ceftiofur, and tetracycline, resistant to enrofloxacin, gentamicin, and neomycin, and showed intermediate susceptibility to sulfamethoxazole/trimethoprim. *K. pneumoniae* ATCC 13883 was resistant to ampicillin and susceptible to all other tested antimicrobials.

*S. aureus* ATCC 25923 demonstrated resistance to enrofloxacin, oxacillin, and sulfamethoxazole/ trimetho-prim, with intermediate susceptibility to gentamicin, neomycin, and tetracycline. The methicillin-resistant *S. aureus* ATCC 43300 strain exhibited resistance to ampicillin, ceftiofur, gentamicin, neomycin, penicillin, and oxacillin, confirming an MDR phenotype.

The clinical isolate of *S. agalactiae* showed resistance to gentamicin, neomycin, oxacillin, and sulfamethoxazole/trimethoprim, with intermediate susceptibility to enrofloxacin, supporting its classification as MDR. In contrast, *S. uberis* remained susceptible to most tested antimicrobials, with intermediate susceptibility observed only for enrofloxacin.

### *In vitro* efficacy of EOs against bacteria associated with mastitis

The MIC and MBC values of *M. alternifolia* and *C. officinalis* EOs are presented in Table 3. *M. alternifolia* inhibited the growth of *E. coli* ATCC 25922 (MIC = 10 mg/mL), with bactericidal activity observed at the same concentration (MBC = 10 mg/mL, MBC/MIC = 1). Inhibitory activity was also detected against the clinical isolates of *S. uberis* (MIC = 15 mg/mL) and *S. agalactiae* (MIC = 31 mg/mL); however, MBC values (125–250 mg/mL) resulted in MBC/MIC ratios > 4, indicating predominantly bacteriostatic effects under the tested conditions. Limited inhibitory activity was observed against *S. aureus* ATCC 25923 (MIC = 25 mg/mL), without bactericidal effect. No inhibitory activity was detected against *K. pneumoniae* ATCC 13883 or methicillin-resistant *S. aureus* ATCC 43300 at the tested concentrations.

**Table 3:** MIC and MBC of *Copaifera officinalis* and *Melaleuca alternifolia* essential oils against bacteria associated with bovine mastitis.

Bacterial strain	EO *C. officinalis* MIC (mg/mL)	EO *C. officinalis* MBC (mg/mL)	EO *M. alternifolia* MIC (mg/mL)	EO *M. alternifolia* MBC (mg/mL)
*Escherichia coli* ATCC 25922	ND	ND	10	10
*Klebsiella pneumoniae* ATCC 13883	ND	ND	ND	ND
*Staphylococcus aureus* ATCC 25923	125	ND	25	ND
*S. aureus* ATCC 43300	250	ND	ND	ND
*Streptococcus uberis*	10	125	15	125
*S. agalactiae*	7.5	ND	31	250

ND = No antimicrobial activity detected at the highest tested concentration (250 mg/mL), MIC = Minimum inhibitory concentration, MBC = Minimum bactericidal concentration, EO = Essential oil

*C. officinalis* exhibited lower antibacterial activity overall. Growth inhibition was observed against *S. uberis* (MIC = 10 mg/mL) and *S. agalactiae* (MIC = 7.5 mg/mL). However, MBC values were either substantially higher than MIC values or not achieved at the highest tested concentration (250 mg/mL), resulting in MBC/MIC ratios > 4 and indicating absence of bactericidal activity under the experimental conditions. No inhibitory activity was detected against *E. coli*, *K. pneumoniae*, or *S. aureus* strains within the tested concentration range.

Overall, *M. alternifolia* demonstrated broader inhibitory activity than *C. officinalis*, particularly against Gram-positive mastitis-associated clinical isolates.

## DISCUSSION

### MDR patterns and clinical relevance

The emergence of MDR pathogens in bovine mastitis represents a growing therapeutic challenge, reinforcing the need to investigate alternative or adjunct antimicrobial strategies. In the present study, the methicillin-resistant *S. aureus* ATCC 43300 strain demonstrated resistance to multiple antibiotic classes, confirming its MDR phenotype and supporting concerns regarding limited therapeutic options in dairy systems. Similarly, the clinical isolate of *S. agalactiae* exhibited multidrug resistance, consistent with reports of resistant streptococcal strains in Brazilian dairy herds [11].

### Antibacterial activity of EOs

EOs have been proposed as potential alternatives due to their complex chemical composition and multimodal mechanisms of action. Among the tested oils, *M. alternifolia* demonstrated broader antibacterial activity, particularly against streptococcal isolates. However, bactericidal activity (MBC/MIC ≤ 4) was observed only against *E. coli* ATCC 25922. For *S. uberis* and *S. agalactiae*, MBC/MIC ratios exceeded 4, indicating predominantly bacteriostatic effects under the tested conditions. This distinction is important for interpreting the translational potential, as bacteriostatic activity may still contribute to therapeutic efficacy, particularly in intramammary infections where host immune mechanisms assist bacterial clearance or when combined with conventional antimicrobials. Moreover, growth inhibition observed *in vitro* does not necessarily imply effective bacterial eradication, highlighting the relevance of pharmacodynamic interpretation.

### Comparison with previous studies

The MIC values observed for *M. alternifolia* (modal values ranging from 10–31 mg/mL) were higher than those reported in some previous studies evaluating tea tree oil against mastitis-associated pathogens, which ranged approximately from 0.78 to 25 mg/mL under *in vitro* conditions [12, 13]. Differences in MIC values may be attributed to several factors, including strain variability, chemotype composition, geographic origin of the EO, and methodological differences such as emulsification procedures and broth composition. The present study evaluated clinical isolates obtained from dairy herds, which may exhibit greater phenotypic resilience compared to laboratory-adapted reference strains. Additionally, variations in terpinen-4-ol concentration and the relative abundance of monoterpenes may influence antibacterial potency.

### Limited activity of *C. officinalis*

*C. officinalis* EO exhibited more limited antibacterial activity. Inhibitory effects were detected against *S. uberis* and *S. agalactiae*, but bactericidal activity was not achieved at the tested concentrations. Previous studies have reported variable antimicrobial activity for copaiba oils, often influenced by the predominance of sesquiterpenes such as β-caryophyllene [14, 15]. In contrast, the EO evaluated in this study was characterized by higher proportions of α-pinene, β-thujene, and D-limonene, which may partly explain differences in antibacterial performance compared to other reports.

### Gram-negative resistance mechanisms

Among Gram-negative strains, *M. alternifolia* inhibited *E. coli* ATCC 25922 but showed no activity against *K. pneumoniae* ATCC 13883. The intrinsic structural barriers of Gram-negative bacteria, particularly the lipopolysaccharide-rich outer membrane, may reduce EO penetration [16]. Additionally, the presence of a polysaccharide capsule in *K. pneumoniae* may further limit susceptibility [17]. Such variability underscores the importance of species-specific evaluation when considering plant-derived antimicrobials.

### Methodological considerations

The methodology followed CLSI recommendations for disk diffusion testing, including blood supplemen-tation for streptococcal isolates. For broth microdilution assays, CAMHB without blood supplementation was used, as commonly adopted in antimicrobial screening studies involving natural products. Although blood supplementation may be recommended in certain clinical MIC determinations for streptococci, the use of unsupplemented CAMHB allows standardized comparison across bacterial species and minimizes potential interference with EO dispersion and resazurin-based viability assessment. Therefore, the results should be interpreted within the context of exploratory *in vitro* screening rather than formal clinical breakpoint determination.

### Limitations of the study

Some limitations should be acknowledged. First, molecular confirmation of the clinical streptococcal isolates was not performed; identification relied on standardized biochemical profiling performed in a reference veterinary diagnostic laboratory with established quality control procedures. Second, the study was limited to *in vitro* antibacterial assays, and the relatively high concentrations required for inhibition raise considerations regarding formulation feasibility, tissue tolerance, and potential cytotoxicity in intramammary applications. Cytotoxicity and selectivity assays using mammary epithelial cell models are currently being conducted as part of an ongoing research line to further evaluate the translational potential of these EOs. Strategies such as nanoemulsion systems or combination approaches may also enhance antibacterial performance while reducing required concentrations [18, 19].

### Implications and future directions

To our knowledge, few studies have directly compared these two EOs under identical experimental conditions against clinically characterized MDR streptococcal isolates from Brazilian dairy herds. Overall, the findings indicate that *M. alternifolia* EO exhibits broader antibacterial activity than *C. officinalis* under the tested conditions, particularly against streptococcal mastitis pathogens. Although bactericidal effects were limited, the observed inhibitory activity supports continued investigation into optimized formulations and combinatory strategies for mastitis control.

Future investigations should also explore potential synergistic interactions between EOs and conventional antimicrobials, as well as antibiofilm activity against mastitis-associated pathogens.

## CONCLUSION

The present study demonstrated that MDR phenotypes were evident in both methicillin-resistant *S. aureus* ATCC 43300 and the clinical isolate of *S. agalactiae*, confirming the growing therapeutic challenge associated with bovine mastitis pathogens. Among the tested EOs, *M. alternifolia* exhibited comparatively broader antibacterial activity, with effective inhibition of *E. coli* ATCC 25922 (MIC = 10 mg/mL; MBC/MIC = 1) and measurable inhibitory effects against *S. uberis* and *S. agalactiae*. However, bactericidal activity was largely restricted, as indicated by MBC/MIC ratios > 4 for streptococcal isolates, suggesting predominantly bacteriostatic action. In contrast, *C. officinalis* demonstrated limited antibacterial efficacy, with inhibitory activity confined to streptococcal isolates and no bactericidal effects observed within the tested concentration range. No activity of either EO was detected against *K. pneumoniae* or methicillin-resistant *S. aureus*, highlighting variability in susceptibility among Gram-negative and MDR pathogens.

A major strength of this study lies in the use of clinically relevant mastitis isolates alongside reference strains, providing a more realistic assessment of antimicrobial performance under field-relevant conditions. The standardized comparative evaluation of two EOs under identical experimental conditions further strengthens the reliability and interpretability of the findings. Additionally, the integration of MIC and MBC assessments enabled a clear distinction between inhibitory and bactericidal effects, contributing to a more nuanced understanding of EO activity.

In conclusion, *M. alternifolia* demonstrated superior antibacterial potential compared with *C. officinalis*, particularly against Gram-positive mastitis-associated pathogens, although its activity was predominantly bacteriostatic. These findings support the potential role of EOs as adjunct antimicrobial agents rather than standalone therapeutics in mastitis management. The variability in efficacy across bacterial species and the relatively high concentrations required underscore the need for formulation optimization and combinatory strategies. Overall, this study provides valuable evidence supporting the continued exploration of plant-derived antimicrobials as part of integrated approaches to address MDR in bovine mastitis.

## DATA AVAILABILITY

The supplementary data can be made available from the corresponding author upon request.

## AUTHORS’ CONTRIBUTIONS

MCB: Methodology, investigation, formal analysis, validation, and drafted the manuscript. MKF: Supervision, resources, project management, data curation, conceptualization, and revised the manuscript. IES: Resources and project management. MS: Investigation and formal analysis. KAB: Investigation, formal analysis, and supervision. All authors have read and approved the final version of the manuscript.
